# Useless Hearing in Male *Emblemasoma auditrix* (Diptera, Sarcophagidae) – A Case of Intralocus Sexual Conflict during Evolution of a Complex Sense Organ?

**DOI:** 10.1371/journal.pone.0087211

**Published:** 2014-01-28

**Authors:** Reinhard Lakes-Harlan, Thomas deVries, Heiko Stölting, Andreas Stumpner

**Affiliations:** 1 Justus-Liebig-Universität Gießen, Institute for Animal Physiology, AG Integrative Sensory Physiology, Gießen, Germany; 2 Georg-August University Göttingen, Cellular Neurobiology, Schwann-Schleiden-Forschungszentrum, Göttingen, Germany; Universität Bielefeld, Germany

## Abstract

Sensory modalities typically are important for both sexes, although sex-specific functional adaptations may occur frequently. This is true for hearing as well. Consequently, distinct behavioural functions were identified for the different insect hearing systems. Here we describe a first case, where a trait of an evolutionary novelty and a highly specialized hearing organ is adaptive in only one sex. The main function of hearing of the parasitoid fly *Emblemasoma auditrix* is to locate the host, males of the cicada species *Okanagana rimosa*, by their calling song. This task is performed by female flies, which deposit larvae into the host. We show that male *E. auditrix* possess a hearing sense as well. The morphology of the tympanal organ of male *E. auditrix* is rather similar to the female ear, which is 8% broader than the male ear. In both sexes the physiological hearing threshold is tuned to 5 kHz. Behavioural tests show that males are able to orient towards the host calling song, although phonotaxis often is incomplete. However, despite extensive observations in the field and substantial knowledge of the biology of *E. auditrix*, no potentially adaptive function of the male auditory sense has been identified. This unique hearing system might represent an intralocus sexual conflict, as the complex sense organ and the behavioural relevant neuronal network is adaptive for only one sex. The correlated evolution of the sense organ in both sexes might impose substantial constraints on the sensory properties of the ear. Similar constraints, although hidden, might also apply to other sensory systems in which behavioural functions differ between sexes.

## Introduction

Adaptive phenotypes are a result of evolutionary processes underlying natural selection, genetic drift and sexual selection. In sexual reproducing species, such phenotypes have also to be balanced between the sexes [1], [2]. Females and males phenotypically differ in many traits, which are regulated by sex-biased genes [3], [4]. This bias may lead to intralocus sexual conflicts (IASC), when a trait in one sex affects the phenotypic optimum in the other sex [5]. Here we investigate a complex sense organ, the ear of flies, in respect to sexual differences and adaptive functions.

In insects hearing evolved several times independently [6]–[9] driven by three basic selection forces: intraspecific communication (like mate finding and spacing, in many species of Orthoptera and Homoptera), predator avoidance (most prominently in nocturnal Lepidoptera to avoid echolocating bats) and host finding (used by a few parasitoid Diptera). Hearing organs may also serve more than one function: for example, crickets use a low frequency band for intraspecific communication and a high frequency band for predator avoidance [10]. If the selection pressure is not the same for both sexes IASC can occur and/or hearing organs might evolve to be sexually dimorphic. Examples of dimorphic ears are found in several groups. For example, mantid ears may show anatomical as well as physiological differences [11], [12]. In mantid species with sexual differences, females have reduced hearing capabilities, especially in the ultrasonic range and the trait correlates with wing reduction. Sexually dimorphic ears have also been described in parasitoid fly species (Ormiini, Tachinidae) [13]–[16]. These flies are parasitoids of nocturnally calling Orthoptera. Host finding is a function of hearing, which is performed only by one sex, the females. The male ear is smaller, is differently tuned and less sensitive when compared to the female ear) [13]–[16]. In Ormiini, the male hearing organ is believed to function for predator ( = bat) avoidance.

In addition to the Tachinidae, some species of Sacrophagidae convergently evolved auditory host finding [17]. The sarcophagid *Emblemasoma auditrix* parasitizes sound producing males of the cicada *Okanagana rimosa* [17], [18]. Like in the Ormiini this parasitoid uses acoustic cues for host localisation, but – as the host - it is active during daylight only and therefore no predator pressure by bats exists. Gravid females perform phonotaxis to the calling song of *O. rimosa* for larva deposition into the host. Here, we investigate whether males have an auditory sense and whether the sense organ is functional physiologically and behaviourally. The findings are discussed in respect to the character evolution in both sexes.

## Methods

Animals were observed and collected in the field near Grayling or Pellston, Michigan, USA. For electrophysiology pupae were collected from infected host cicadas (*Okanagana rimosa)*. The pupae were transferred to University of Göttingen, Germany and kept for up to six months at 4°C. Adult flies of both sexes emerged 14 to 20 days after transferring them into room temperature. Adult flies were kept with water and sugar *ad libidum* and were investigated 3–5 days after emergence.

In the field, male *E. auditrix* were collected by sweeping the vegetation and were transferred to the Biological Station of the University of Michigan (UMBS), Pellston for behavioural tests. Animals had their wings clipped off to prevent escape and were kept in small cages, with sugar and water *ad libidum*. Flies were tested up to seven days after their capture. Altogether 17 flies were tested, up to three times in an experiment.

The arena for behavioural tests (50 cm x 70 cm) was weakly illuminated from above (300–430 Lux). The arena was covered with cloth to minimise optical cues. One piezo loudspeaker (HT-Horn; Conrad Electronic) was placed behind the cloth in the centre of one side. Flies were released in 50 cm distance from the front of the loudspeaker. For phonotaxis experiments the calling song of the host *O. rimosa* was digitised (from a mastertape of T. Moore, Ann Arbor, USA) and stored on a compact disc (CD, 44.1 kHz sampling rate). The calling song consisted of chirps with 8–10 kHz peak frequency, 6 ms duration and a repetition rate of 83 chirps per second (cps); cicadas emit the calling song with 90 dB SPL at 10 cm distance [19]. Song models of the calling song were created using CoolEdit (Syntrillium Coop.) and also stored on compact disc. The first model had a carrier frequency of 9 kHz, a repetition rate of 80 cps and comprised two pulses (one of 1 ms and one of 4 ms separated by 1 ms pause, for a total duration of 6 ms, resembling the temporal structure of the calling song). The second model had a carrier frequency of 5 kHz corresponding to the best hearing frequency. The signals were played back with a discman (Sony D-131) connected to a custom built amplifier and attenuator in order to adjust sound intensity at the release point of the flies. Sound intensity was measured using a sound level meter (Bruel & Kjael 2203) equipped with a 1/2“ microphone (B & K 4165). The intensity was varied in steps of 5 dB between 60 and 85 dB SPL (rel. 20 µPa). A discriminative scoring system was developed in order to detect minute behavioural elements of phonotaxis. The behaviour was observed and scored in three classes: class 1 - turns towards the loudspeaker, class 2 - phonotaxis of 20 cm, class 3 - complete phonotaxis (50 cm). For statistical analyses each class was assigned one point and the mean number of points was calculated. Statistical analysis included contingency table tests and ANOVA using Prism software (GraphPad Coop.).

In the field the signals were broadcasted with a sound pressure level of 90–100 dB SPL and animals performing phonotaxis were collected [20]. Acoustic attraction experiments were also made at clearings with dry vegetation, where male flies had been caught by sweeping with a net. At these places sounds have been recorded on digital recorder (Tascam DR-100, 44.1 kHz cut off frequency) to identify any specific sounds males are exposed to.

For electrophysiological determination of the hearing threshold the animal was fixed dorsal side up and the neck connective was exposed [18]. The hearing threshold was determined by suction electrode recordings from axons of auditory interneurons in the neck connective. Sound stimuli (comprising pure sine waves between 3 and 50 kHz and a duration of 50 ms with pauses of 250 ms) were synthesised with a PC-controlled sound board and presented with a single speaker. For other details of the electrophysiological setup see [15], [16].

For scanning electron microscopy of the tympanal organ, the anterior thorax was dissected and fixated in 4% paraformaldehyde. After dehydration, preparations were critical point dried (Balzers, CPD 030) and sputtered with gold (Baltec SCD 050). The preparations were viewed with a Leo 438VP scanning electron microscope and pictures were digitised (1024×768 pixel) with a CCD camera. Additional morphometric measurements were performed with a dissecting microscope (Leica MS5) and a calibrated ocular.

### Ethics statement

For the field work, access to the Pellston site was permitted by the Biological Station of the University of Michigan (UMBS). The Grayling site is public land, no permission is required. *Emblemasoma auditrix* is not protected and experiments were done in accordance with the regulations for invertebrate research in the US and Germany.

## Results

**Figure 1:**
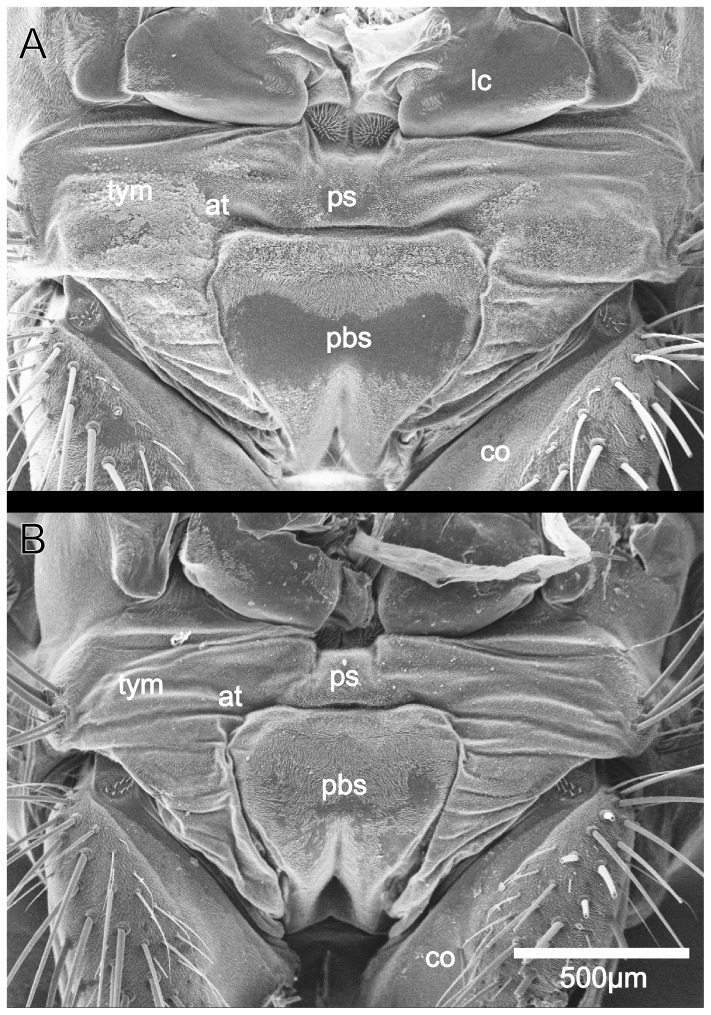
Scanning electon micrographs of the ear of a female (A) and a male (B) *Emblemasoma auditrix*. at: attachment site of the sensory cells, co: coxa of the foreleg, lc: lateral cervicale, pbs: probasisternite, ps: prosternite, tym: tympanal membrane. Dorsal is to the top.

The sarcophagid *Emblemasoma auditrix* possesses an ear at the prothorax, directly behind the head (Fig. 1). The ear is sexually dimorphic with the female ear broader than the male ear (table 1). The ear width, measured from one side to the other side of both tympanal membranes, as well as the width of the probasisternite (a cuticular element ventral of the tympanal membrane) is about 8% larger in females than in males. Head widths of males and females do not differ, whereas males have a longer femur than females (table 1). The ear width correlates best with the head width (females: r = 0.806; males: r = 0.612).

**Table 1:** Mean length and width of different morphological structures of males and females in mm (s.e.m. in parentheses).

	Male (n = 21)	Female (n = 22)	P-value
Femur length	2.73 (0.039)	2.61 (0.023)	P = 0.0241*
Head capsule width	3.15 (0.028)	3.23 (0.031)	P = 0.1003
Ear width	5.38 (0.058)	5.82 (0.055)	P<0.0001****
Probasisternite width	1.94 (0.022)	2.12 (0.049)	P = 0.0021**

Statistics: unpaired t-test with Welch's correction comparison of male and female; the ear width is highly significantly different between males and females.

**Figure 2:**
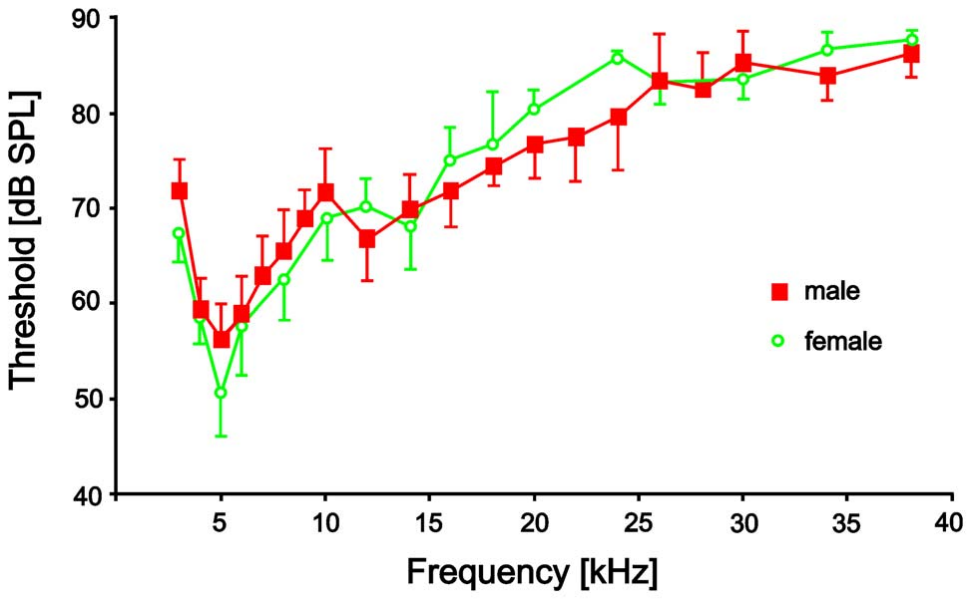
Hearing thresholds (mean and s.e.m.) of female (green) and male (red) *E. auditrix*. The hearing threshold was determined based on extracellular recordings from the neck connective. Hearing threshold for female adapted from [17]. N = 3 for female, N = 4 for male.

The male hearing organ is physiologically functional. The hearing threshold has a minimum at about 5 kHz and is relatively insensitive in the ultrasonic range (Fig. 2). Interestingly, the threshold curves of males and females are rather similar in respect to tuning and general sensitivity.

In the field, female *Emblemasoma auditrix* can be attracted to a loudspeaker broadcasting the calling song of the host. In hundreds of experiments in 12 years of field research only females have been caught on the speaker. In rare cases (less than 5 observations during 12 years) male flies were detected in the vicinity of the experimental setup. More regularly, male flies could be caught early in season by sweeping patches with dry vegetation. At these patches matings have been observed; analyses of the status of mating females showed that they had developed ovaries, but no larvae (n = 3). Recorded sounds at these places included bird vocalizations and environmental noise (wind), but no specific fly related sounds.

**Figure 3:**
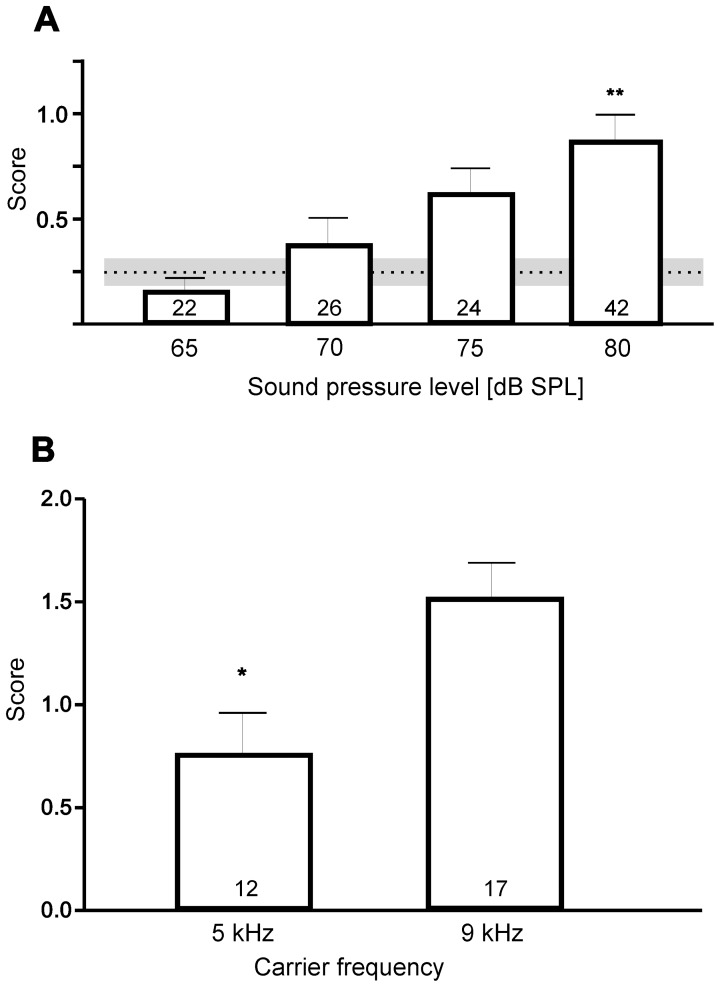
Phonotactic behaviour of male *E. auditrix*. The behaviour was scored in three classes, which were assigned one point each. Class 1: turning towards the speaker, class 2: moving towards the speaker (20 cm), class 3: complete phonotaxis and reaching the speaker (in 50 cm distance). **A** Phonotactic score in respect to the sound pressure level of the calling song. The dashed line indicates the score without sound; the grey area indicates the variation (+- s.e.m.). The numbers of tests are indicated in the columns. Each animal was tested twice (N = 12, n = 24 without sound; N = 11, n = 22 for 65 dB SPL; N = 13, n = 26 for 70 dB SPL; N = 12, n = 24 for 75 dB SPL), except for 80 dB SPL (N = 14, n = 42). Mean with s.e.m., ANOVA with Bonferroni correction. **B** Phonotactic score in respect to song models with different carrier frequencies (both at 80 dB SPL). Despite the more sensitive hearing at 5 kHz, the phonotactic score to 5 kHz models is significantly lower than to 9 kHz models (p = 0.0113, t = 3.27, df =  11, paired t-test). The numbers of tests are given in the columns; each animal was tested once. Mean with s.e.m.

Behavioural experiments in the laboratory clearly demonstrated that male flies react to sound signals. A male was released in the arena and the behaviour in response to the calling song of the cicada was quantified. Complete phonotaxis has not been observed in contrast to females [21]. Therefore, a more discriminative assay than that used for female phonotaxis had to be designed: Points were assigned to three different levels in behaviour (turning towards the speaker, initial directed movement, completed phonotaxis). Control males which were released in the arena without acoustic signals turned towards the loudspeaker only by chance and reached a mean score of 0.25 points (Fig. 3A; s.e.m.: 0.091, n = 24). When stimulated with the calling song of the host about 80% of the animals turned towards the speaker and the behavioural score increased with increasing sound pressure level (Fig. 3A). At 80 dB SPL the score reached nearly the value 1 and was significantly different from the control level; in this scoring system females reach a score of nearly 3 [22]. Due to variability in the male response individual thresholds for single males have not been determined. Additionally, males were tested with a song model with 9 kHz carrier frequency (corresponding to the peak frequency of the host calling song), in comparison to a model with 5 kHz carrier frequency (best hearing frequency). Males showed a frequency dependent behavioural reaction, with higher scores in response to the model with 9 kHz carrier frequency (Fig. 3B). Two out of 17 males even showed a complete phonotaxis to the song model with 9 kHz carrier frequency.

## Discussion

The results clearly show that males of *E. auditrix* possess an ear, are able to hear and may use it for phonotaxis towards cicada songs. Nevertheless, since the known hearing function (host finding for larvae deposition) is exclusively related to the female, the main question arises: why do males hear at all? We propose that for the species *E. auditrix* hearing has an adaptive value for the female only, while for the male no detectable function exists. This proposed non-function in one sex can only be revealed with negative evidence, which principally is difficult to produce. In the following we firstly carefully check the male hearing sense against the known functions of hearing in insects [9], before relating the findings to general questions.

### 1) Function in intraspecific signalling, either between sexes or between individuals of the same sex

This function of insect hearing requires sound production of the species. Evolutionarily, in a taxon like Orthoptera sound production is rather old and it is even more widely distributed than hearing [9], [23], [24]. However, no intraspecific acoustic communication signals are known in *Emblemasoma* and no specialized sound producing structures have been detected on the flies. Flies may produce buzzing noises during flight, but these noises do not seem to play a role for long range intraspecific signalling: no buzz related auditory behaviour has been detected and the rather high hearing threshold makes such behaviour unlikely. For Diptera in general it is known that Near Field sounds produced by the wing movements may be important for sex recognition in short distance and that such sounds contain frequencies below 1 kHz [25]. The antenna reacts to the particle velocity of these sounds (Mosquitos [26]–[28]; Drosophila [29], [30]). By contrast, the tympanal ear of *E. auditrix* is a sound pressure receiver, reacting to Far Field sounds of higher frequencies than 1 kHz.

In addition to self-generated sounds, also sound from external sources might be used by both sexes for intraspecific interaction, like gathering together at a sound source. However, screening for environmental sounds in the habitat did not reveal any abiotic sound source which might attract both sexes. Furthermore, non-biological noises are irregular and have frequency spectra below 2 kHz [31]. A biological sound source with such a function, of course, could be the cicada calling song, which is discussed under function 3.

### 2) Function in predator detection

It is self-evident that it is advantageous to detect all kinds of stimuli, which would indicate appearance of a potential predator. The male ear, therefore, might serve as a predator detector. However, for what predators might the ear be specifically adaptive? *E. auditrix* is active during (sunny) daytime [20] and it is unlikely that bats – a major predator group driving evolution of hearing in insects - predate on them.

In direct contrast to *E. auditrix* hearing tachinid flies are active during night. Therefore, a predator avoidance function has been suggested for male hearing in *Ormia ochracea* [13]. The suggestion is based on the shape of the threshold curve, as well as on the difference to female hearing, but evasive behaviour of males has not yet been shown. Like in *E. auditrix* it never has been observed that males of Ormiini are attracted to songs of hosts (*O. depleta* [32]; *O. ochracea* [33]; *H. alleni* [34]). Little is known about the biology and activity of males and also for potential predator pressures. Male *Ormia* seem to aggregate in large groups on top of exposed landmarks as a waiting station for mating [35]. Non-hearing tachinids show the same behaviour, even at the same sites [35]. This questions the selection pressure for hearing in males at least in this behavioural context. Nevertheless, the hypothesis of bat avoidance in male tachinids is plausible, but it does not apply to the known activity pattern of *E. auditrix*.

Selective pressure for auditory detection of diurnal predators has not been clearly demonstrated, although some butterflies might be able to detect bird sounds [36]. In general, birds, reptiles, or predatory insects might catch *E. auditrix*, but no specific predator (and related sounds) has been detected. For example, wing beat noises have lower frequencies [36] than sensitive hearing in *E. auditrix* (peak at 5 kHz). Additionally, sympatric blow fly species do not possess an ear. A well-developed visual sense and a vibration sense seem to be more important for predator avoidance. Like many other flies, *E. auditrix* reacts sensitive to vibratory stimuli [17] and has prominent eyes.

### 3) Function in host detection

This function is exclusively necessary for females of the investigated parasitoid species. A female is perfectly able to infect a cicada [37], and males are not necessary during the infection process. Could male phonotaxis towards the host calling song be an indirect way of mate finding? The reproductive behaviour of *E. auditrix* does not support such a hypothesis either: Already in early June nearly all female *E. auditrix* which were attracted to the loudspeaker were carrying fully developed larvae in their uterus [22]. Matings must have taken place some days earlier, as larvae need some time to develop. Furthermore, multiple matings are unlikely, since later in the season the ovaries were shrunken in females [22] and mating flies did not carry larvae. Additionally, observations of 12 years field work support the lack of male attraction to the host, as males have never been attracted to a loudspeaker (by contrast to far more than thousand females) and mating has never been observed during the phonotactic experiments.

By contrast to phonotaxis, visually guided male chasing behaviour and mating have been observed in the appropriate microhabitat. For mating male *E. auditrix* follow fast moving objects as do many other Oestroidea flies [38]. Male flies even may possess specific neuronal networks for the chasing behaviour and detection of small objects [39]. Thus, the visual sense probably plays the major role in mate finding.

Furthermore, the reproductive behaviour of this fly species is related to the seasonality of the host. Adults of the host cicada species emerge from the ground by mid-June [19] and it takes some more days before cicada males start calling. Flies are present in the biotope before the host is available. A similar temporal pattern has also been observed in the ormiine fly, *Therobia leonidei* (Lehmann, pers. comm.) and seems to be adaptive to ensure an overlap in seasonality of parasitoid and host. Taken together, mate finding in *E. auditrix* does not rely on the cicada song.

#### Physiology of hearing in both sexes and intralocus sexual conflict

None of the functions suggested so far for insect hearing seems to be relevant for male *E. auditrix*. Whereas the hearing system is clearly present in males, the positive behavioural reaction to the host signal, however, was detected by a sensitive scoring system. In the following we discuss the physiology in respect to genetic links which might have led to the evolution of the sense organ in both sexes, although it is needed only in one sex.

Evolution of traits is balanced between sexes [1]–[3] and intralocus sexual conflict occurs when the expression of sexually antagonistic alleles increases the fitness in one sex, but decreases the fitness in the other sex [4], [5]. Here, conflicting selection pressures might occur for the hearing sense of *E. auditrix* since a probably functionless trait has evolved in the context of correlated characters. Ears in both sexes are rather similar, e.g. in respect to tuning to 5 kHz instead of matching the peak frequency of the host's calling song (9 kHz). This mismatch has already been puzzling during interpretation of the female hearing [17], [20], [21]. For host detection it has been assumed that temporal structures of the calling song are more important than peak frequency [21]. The finding that males have the same best hearing frequency as females indicates the tuning of the ears being a general phenomenon. So far, the reasons for the mismatch can only be speculated upon. Since the ear is also sensitive to substrate vibrations [17], the tuning might be related to that physiological function. Ongoing physiological experiments test the hypothesis that vibration detection is important for both sexes.

A non-functional organ in one sex might also lead to sexually dimorphic sense organs or to reduction of the organ. Both processes are observed among hearing insects. In insect species with sexually dimorphic hearing organs divergent functions for the sexes have been found or proposed [11], [12], [40], [41]. The best investigated taxon in this respect are Mantodea [11], [12]: female mantids in different lineages have reduced their hearing capabilities to different degrees. This reduction is best correlated with a partial or complete reduction in wings and has therefore been related to reduced selection pressure by bats [11]. A correlation of wing loss, correspondingly reduced predator pressure and reduced hearing has also been found in other taxa [40], [42], [43]. In moths a loss of predation may lead to a reduced sensitivity in hearing [44], [45]. Such a regression typically affects both sexes, as in grasshopper which lost acoustic communication [46]. The lack of regression in *E. auditrix* might be due to the evolutionary origin from a precursor chordotonal organ the function of which is still represented in the present ear [17]. Nevertheless, a biological function of hearing in males is missing and genetic coupling might have influenced the evolution.

The developmental genetics for formation of a tympanal ear in insects are not known and hopefully future gene expression studies might reveal the mechanisms for development of the different phenotypes. Nevertheless, possible intralocus sexual conflicts would have implications for interpretation of the traits for hearing and evolution of insect ears in general. For intraspecific acoustic communication, the basic scheme is that one sex (male) signals and the other sex (female) responds to the sounds [9]. Thus, the evolution of an ear might in the first place have been adaptive for females. Because of a genetic linking, the male would have evolved an ear as well and hearing would have acquired secondary functions, like intraspecific spacing of signaling males, duet communication or predator avoidance. Such a view is also supported by the fact, that in some taxa the sound producing structure of females and males evolved independently [47], [48]. These constraints should be kept in mind, when interpreting the physiology of hearing.

Following the arguments above, we have identified a sense organ that is functional in both sexes but is probably not adaptive in males. This process could only be detected in an example, in which despite careful investigations no - not even a speculative - function was detected. It seems plausible that the hearing sense evolved correlated in both sexes, even though it has a function in one sex only. The evolutionary origin of insect hearing organs also speaks for the hypothesis that the initial traits are genetically coupled and that only later the traits came under the influence of sex-specific adaptations.
